# Disease burden, clinical management and unmet treatment need of patients with moderate to severe alopecia areata; consensus statements, insights, and practices from CERTAAE (Central/Eastern EU, Russia, Türkiye AA experts) Delphi panel

**DOI:** 10.3389/fmed.2024.1353354

**Published:** 2024-04-22

**Authors:** Lidia Rudnicka, Magdalena Trzeciak, Erkan Alpsoy, Petr Arenberger, Sibel Alper, Nina Benáková, Svetlana Bobko, Murat Borlu, Magdalena Czarnecka Operacz, Burhan Engin, Tülin Ergun, Ilgen Ertam Sağduyu, Olga Filipovská, Aida Gadzhigoroeva, Martina Kojanová, Aleksandra Lesiak, Anna Michenko, Nikolay Murashkin, Nahide Onsun, Witold Owczarek, Zuzana Plzakova, Adam Reich, Marie Selerová, Burcu Aybike Gürbüz

**Affiliations:** ^1^Department of Dermatology, Medical University of Warsaw, Warsaw, Poland; ^2^Department of Dermatology, Venereology and Allergology, Medical University of Gdańsk, Gdańsk, Poland; ^3^Department of Dermatology and Venereology, Akdeniz University School of Medicine, Antalya, Türkiye; ^4^Department of Dermatovenerology, Third Faculty of Medicine, Charles University, Prague, Czechia; ^5^Department of Dermatology and Venereology, Koç University, İstanbul, Türkiye; ^6^Department of Dermatovenereology, First Faculty of Medicine, Charles University, Prague, Czechia; ^7^Moscow Scientific and Practical Centre of Dermatovenereology and Cosmetology, Moscow, Russia; ^8^Dermatology and Venereology Department, Erciyes University, Kayseri, Türkiye; ^9^Department of Dermatology, Medical University of Poznań, Poznań, Poland; ^10^Department of Dermatology and Venerology, Cerrahpaşa Medical Faculty, Istanbul University-Cerrahpaşa, İstanbul, Türkiye; ^11^Department of Dermatology, Marmara University, İstanbul, Türkiye; ^12^Department of Dermatology, Ege University, Izmir, Türkiye; ^13^Department of Dermatology, Masaryk Hospital in Ústí nad Labem, Ústí nad Labem, Czechia; ^14^Moscow Scientific and Practical Center of Dermatovenereology and Cosmetology, Moscow, Russia; ^15^Laboratory of Autoinflammatory, Genetic and Rare Skin Disorders, Department of Dermatology, Pediatric Dermatology and Oncology, Medical University of Lodz, Lodz, Poland; ^16^Federal State Budgetary Institution of Continuing Professional Education, Central State Medical Academy, Moscow, Russia; ^17^Medical Research and Educational Center, Lomonosov Moscow State University, Moscow, Russia; ^18^National Medical Research Center for Children’s Health, Moscow, Russia; ^19^Sechenov First Moscow State Medical University (Sechenov University), Moscow, Russia; ^20^Dermatology Department, Federal State Autonomous Institution, Scientific Centre of Children’s Health of the Ministry of Health of the Russian Federation, Moscow, Russia; ^21^Research Institute for Pediatrics and Children’s Health Protection, Federal National Public Healthcare Institution “Central Clinical Hospital of the Russian Academy of Sciences”, Ministry of Science and Higher Education, Moscow, Russia; ^22^Dermatology Department, Bezmialem Vakıf University, İstanbul, Türkiye; ^23^Department of Dermatology, Military Institute of Medicine, Warsaw, Poland; ^24^Department of Dermatology, University of Rzeszów, Rzeszów, Poland; ^25^Department of Dermatology, Hospital AGEL Novy Jicin, JSC, Novy Jicin, Czechia; ^26^Medical Affairs, Pfizer, İstanbul, Türkiye

**Keywords:** alopecia areata, disease burden, diagnosis, treatment, clinical management

## Abstract

**Objectives:**

This study aims to update the understanding of Alopecia Areata (AA) in Poland, Czechia, Russia, and Türkiye, focusing on the disease burden, clinical management, and patient journey. It seeks to establish a consensus on optimal management strategies for AA in these regions.

**Methods:**

A modified 2-round Delphi panel was conveyed with 23 Dermatologists (Russia; 4, Türkiye; 7, Poland; 6, and Czechia; 6). The Delphi questionnaire consisted of 61 statements and 43 questions designed to obtain an overall understanding of the perception and acceptance of available information regarding the care of patients with alopecia areata.

**Results:**

The study revealed that moderate-to-severe AA significantly impacts patients’ and their families’ QoL, consistent with previous studies. AA was found to cause more substantial impairment when additional lesions appeared in visible areas besides the scalp. Work and productivity impairment were notably higher in adults with moderate-to-severe AA. Diagnostic consensus highlighted the importance of skin biopsies and trichoscopy, while the need for more practical severity scoring systems was emphasized. Current treatments, including topical therapies, corticosteroids, and systemic immune modifiers, were deemed insufficient, highlighting the unmet medical need.

**Conclusion:**

The Delphi study underscores a significant disease burden and unmet medical needs in patients with moderate-to-severe AA. It highlights the necessity of access to novel treatments and further research to develop more effective therapies with a tolerable safety profile. The findings align with global research, emphasizing the psychosocial impact of AA and the need for standardized, effective treatment protocols.

## 1 Introduction

Alopecia areata (AA) is a common hair loss disorder with a wide range of clinical presentations. Although AA affects approximately 2% of the general population with an unpredictable clinical course and sudden relapse at any given time, robust and recent epidemiology data for AA are still lacking (1, 2). Current evidence suggests that the cause of the condition is autoimmune, with a significant genetic contribution, which is further influenced by unknown environmental factors. Reported triggers include emotional or physical stress, vaccinations, viral infections, and medications (3). While some patients experience spontaneous recovery, others progress to more extensive hair loss, including alopecia totalis and alopecia universalis, with a low chance of full recovery (4).

Current treatment options for AA include topical and intralesional corticosteroids, topical immunotherapy, and oral immunomodulators. However, the efficacy of these treatments varies, and there is no clear consensus on the optimal treatment pathway for AA (3, 5). In recent years, Janus kinase inhibitors (JAK inhibitors) have shown promise in the treatment of AA, with several studies reporting positive outcomes. However, there are concerns about the potential side effects and long-term safety of these drugs (5). Furthermore, the psychological impact of AA cannot be underestimated, as it can be a disfiguring disease with no available cure or therapies to prevent disease relapse.

While data on the global burden of AA are scarce, the disease has been shown to have a substantial impact on quality of life (QoL) (1). Disease management patterns for AA vary across countries; therefore, there is a need for a more comprehensive understanding of the disease burden, clinical management, and multiple aspects of the journey of patients with AA in different countries and regions (3, 5). This study aims to update our understanding of AA in Poland, Czechia, Russia, and Türkiye by obtaining information-driven insights from expert physicians regarding the current disease burden, clinical management, and multiple aspects of patients’ journey with AA. Ultimately, this study seeks to establish a consensus on the optimal management of AA in these regions.

## 2 Methods

### 2.1 Study design

We used a Modified Delphi Method for executing this study. The modified Delphi Method is a research technique that aims to achieve a consensus among a group of experts on specific issues. This method involves multiple rounds of surveys and discussions, typically over several months. The Delphi method is beneficial when limited data is available or when knowledge is uncertain or incomplete. There are no set rules for selecting the appropriate number of Delphi members or rounds. Still, generally, a classic Delphi strategy involves three or more rounds, while modified versions may be completed in just two rounds (6, 7).

The modified Delphi method used in this study consisted of two rounds of online surveys conducted over 2 years (between November 26, 2020, and November 2022). During each round, panel members were able to revise their earlier answers based on the responses of others. This modified method allowed for a literature review and multiple online surveys to transform expert opinions into a consensus-based group decision. The questionnaires used in this modified Delphi method can be obtained from corresponding author upon request.

### 2.2 Identification of participants

At the outset of the study, a scientific committee consisting of four experienced dermatologists was formed, with one member acting as the study coordinator and three other members selected from Czechia, Poland, Türkiye, and Russia, respectively. The study coordinator oversaw the study’s design and progress, including data analysis and manuscript execution.

To ensure the study’s topics were adequately addressed, an independent expert consultant facilitated a discussion among the scientific committee members, who then selected and approved the relevant issues based on the limited world evidence available.

To participate in the study, candidates were selected based on their specific interests and extensive experience in alopecia areata. A total of 23 Dermatology specialists from universities and public hospitals in Russia, Türkiye, Poland, and Czechia were chosen, each with at least 10 years of experience with alopecia areata. Each panelist also had a proven track record in contributing to the field of alopecia areata, either by serving as a board member of an academic association, contributing to guideline development, or publishing articles on the topic. Finally, 22 participants equally contributed to the manuscript development and validated the final version. The independent expert consultant did not get involved in the manuscript development and she did not want to be included in the authors list. Due to a health problem, one participant could not participate in the second round and the validation of the final manuscript.

### 2.3 The formulation of questions and assessment of answers

Before conducting the first round of the study questionnaire, the same independent consultant mentioned in the participant identification part also conducted a comprehensive literature review. The consultant searched for relevant articles published between 2010 and 2020 in several databases, including MEDLINE, Web of Science, Google Scholar, and EMBASE. The search terms used were focused on various aspects of alopecia areata, such as its burden, pathogenesis, etiology, diagnosis, severity, treatment, and response to treatment. The literature review also identified evidence-based recommendations and care pathways as selection criteria. Additionally, relevant guidelines published between 2010 and 2020 were systematically reviewed to ensure the study questionnaire aligned with current best practices. The consultant then prepared the survey questions to avoid participation bias among panelists. Based on the main topics identified in the literature review and guidelines, a total of 133 questions were developed for the study questionnaire, covering different aspects of alopecia areata, such as disease burden, diagnosis, severity, treatment, treatment response, treatment landscape, and unmet medical needs.

The questions were designed using a 5-point Likert response scale or multiple-choice answers with an additional open-ended choice to capture the panel members’ opinions. An electronic questionnaire was used to collect the panel members’ responses, and a consensus/dominant approach was achieved when 70% of the panel members strongly agreed or agreed (or strongly disagreed or disagreed) with a statement or selected the same answer. Statements with less than 40% agreement were dropped from the second Delphi round and not repeated.

Second round of the study delayed for a year and a half due to some of the participants’ serious health problems and several other logistic issues. The questions which were identified to be asked again at the second round were checked by the independent consultant to see if there has been any new significant developments in the evidence over the last year which might affect the validity of these questions. No significant change has been observed, therefore, identified questions were repeated, either using the same question or rephrased content based on the commentaries/corrections made by the participants during the first round. Inconsistencies between different countries were recognized as a non-consensus factor. This process ensured that the study questionnaire was designed based on the latest evidence and was aligned with current best practices.

## 3 Results

### 3.1 Questionnaire structure and general results

The study questionnaire comprised five distinct sections, which aimed to evaluate various aspects of alopecia areata, such as disease burden, diagnosis, disease severity, treatment, treatment response, and unmet treatment needs. The questionnaire consisted of 61 statements and 43 questions designed to obtain an overall understanding of the perception and acceptance of available information regarding the care of patients with alopecia areata in the four countries included in the study.

All participants responded to all questions in the first round; however, one could not answer the second-round survey due to health problems. The consensus was reached on 53 of the 61 statement questions. The level of agreement ranged between 79 and 100%, with the highest consensus percentages observed in the diagnosis and disease severity sections and the lowest in the treatment section. Regarding the remaining 43 questions, the most common observations, perspectives, and clinical practices were reported in the disease burden and diagnosis. Furthermore, it was observed that the clinical practices for assessing disease flare, treatment response, and treatment preferences varied significantly between countries and even within each country (Table 1).

**Table 1:** General results.

Statement sections	Total statement numbers in each section	Consensus in each section n (%) *
A. Disease burden	6	5 (83)
B. Diagnosis	12	12 (100)
C. Disease severity	6	6 (100)
D. Treatment	24	19 (79)
E. Disease flare, treatment response, unmet need	13	11 (84)

Common observations, perspectives, and practices*		
*Survey sections*	*Questions asked in each section*	Common perspectives and practice*
*1. Disease burden*	*7*	*7 (100)*
*2. Diagnosis*	*9*	*9 (100)*
*3. Disease severity*	*4*	*2 (50)*
*4. Treatment*	*15*	*8 (53)*
*5. Disease flare, treatment response, unmet need*	*8*	*3 (37)*

*More than 70% of the total participants and the participants in each country.

### 3.2 Disease burden

The consensus was achieved on most of the statement items in this section. The highest consensus was the ‘Moderate-to-severe AA causes stigmatization in society.’ statement (100%). Participants agreed on the fact that all forms of AA cause considerable psychological disorders, while moderate-to-severe AA causes significant psychological disorders and results in a dramatic impact on the QoL for patients and their families (86.9, 91.2%). They also reported that additional lesions in visible areas beside the scalp (eyebrows, eyelashes, beard, etc.) impair the patient’s QoL to a greater extent (95.6%).

Despite participants agreed on that AA is perceived as an impairing and disabling disease in their societies, they also stated that AA-related impairments and disabilities are still not adequately publicized/emphasized enough in media to raise public awareness of the AA disease burden (86.9, 81.8, 78.2%). Additionally, QoL indexes and psychiatric consultations were not found to be performed as much as they should evaluate AA patients in daily practice (100, 81.8%) (Table 2).

**Table 2:** Results regarding AA disease burden perception.

Statements*	Consensus %
Disease burden	
All forms of AA cause considerable psychological disorders (such as anxiety and mood disorders) and lower the QoL for patients and their families.	86.9
Moderate-to-severe AA causes significant psychological disorders and results in a dramatic impact on the QoL for patients and their families.	91.2
Moderate-to-severe AA causes stigmatization in society.	100
Additional lesions in visible areas beside the scalp (eyebrows, eyelashes, beard, etc.) impair the patient’s QoL to a greater extent.	95.6
Moderate-to-severe AA causes significant work and productivity impairment in adults.	82.5
AA is an impairing disease.	95.6

Common observations, perspectives, and practices*	*%*
*Disease burden*	
*The rate of referring to a healthcare provider is increased in AA patients compared to the general population without AA.*	*78.2*
*AA is perceived as an impairing disease in our society.*	*86.9*
*AA is perceived as a disabling disease in our society.*	*81.8*
*AA-related impairments and disabilities are not adequately publicized/emphasized enough in media in order to raise public awareness of the AD disease burden.*	*78.2*
*Clinical practice*	
*I take into consideration the patient’s QoL when making treatment decisions.*	*85.5*
*QoL indexes are not used as much as they should be to evaluate AA patients in daily practice in my country.*	*100*
*Psychiatric consultations for patients with moderate to severe AA are not performed much as they should evaluate AA patients in daily practice in my country*	*81.8*

*More than 70% of the total participants and of the participants in each country. AA, alopecia areata.

### 3.3 Diagnosis and patient journey

The consensus was achieved in all the statement items, and participants were also reported to be very similar regarding their observations, perspectives, and clinical practices for the diagnosis of AA (Table 3). The consensus with the highest percentages was ‘In certain situations, skin biopsies should be considered to exclude other conditions (e.g., when the cause of the hair loss is unclear),’ ‘Autoimmune comorbidities (such as vitiligo, systemic lupus erythematosus, psoriasis, inflammatory bowel disease, thyroid disorders, ocular diseases, and rheumatoid arthritis) are more common in AA patients. Compared to the general population without AA.’ (100, 95.6%).

**Table 3:** Results regarding AD diagnostic approach and patient journey.

Statements*	Consensus %
Diagnosis	
AA requires a multidisciplinary (such as pediatrics, dermatology, immunology, endocrinology, psychiatry, etc.) approach regarding the diagnosis of the condition.	81.8
Hair-pull test positivity helps distinguish active disease in alopecia areata.	78.2
Hair-pull test positivity in clinically uninvolved areas is an important sign of progressive disease.	78.2
Trichoscopy is the most important tool for the diagnosis of AA.	82.5
Trichoscopy is essential in the assessment of disease activity and severity as well as therapeutic monitoring of AA	82.5
The trichogram is only a useful complementary tool for clinical evaluation, diagnosis, and the monitoring of treatment response.	81.8
In certain situations, skin biopsies should be considered to exclude other conditions (e.g., when the cause of hair loss is unclear).	100
The rationales and criteria for performing a biopsy for AA diagnosis are not well established.	86.3
Autoimmune comorbidities (such as vitiligo, systemic lupus erythematosus, psoriasis, inflammatory bowel disease, thyroid disorders, ocular diseases and rheumatoid arthritis) are more common in AA patients. Compared to the general population without AA	95.6
Neuro-psychiatric comorbidities (such as epilepsy, depression, migraines, attention deficit, and bipolar disorders) are more common in AA patients compared to the general population without AA.	72.7
Neuro-psychiatric comorbidities are more frequently observed in adult AA patients than pediatric AA patients.	77.2
Currently, there are no validated biomarkers that aid in the diagnosis of AA	86.9

Common observations, perspectives, and practices*	*%*
*Diagnostic journey*	
The specialty/ies, children with AA (families) most commonly referred to with their first symptoms: Dermatology**(Experts from Russia, Poland, and Czechia reported the same rate for Dermatology and Pediatrics)	*86.9*
The specialty/ies, adult patients with AA most commonly refer to with their first symptoms: Dermatology**	*100*
*The specialty/ies, child patients with AA most commonly have their diagnosis at Dermatology*	*95.6*
*The specialty/ies, adult patients with AA most commonly have their diagnosis at Dermatology*	*100*
The average time for AA patients to reach a diagnosis from their first symptom: is < 6 months**(Russia < 3 months)	*82.6*
*Clinical practice*	
*I use trichoscopy to examine my patients with moderate-to-severe AA.*	*78.2*
*I investigate autoimmune comorbidities (such as vitiligo, systemic lupus erythematosus, psoriasis, inflammatory bowel disease, thyroid disorders, ocular diseases and rheumatoid arthritis…) in my adult patients with moderate to severe AA.*	*86.9*
*I investigate autoimmune comorbidities in my pediatric patients with moderate to severe AA.*	*86.9*

*More than 70% of the total participants and of the participants in each country. **Variation between countries, the total response is still over 70%. AA, alopecia areata.

They agreed that trichoscopy is essential in the assessment of disease activity and severity as well as therapeutic monitoring of AA, whereas trichogram was found to be only a useful complementary tool for clinical evaluation, diagnosis, and the monitoring of treatment response (82.5, 81.8%). The vast majority of the participants stated that they use trichoscopy to examine patients with moderate-to-severe AA (78.2).

### 3.4 Disease severity

The consensus was achieved in all the statement items in this section. The highest consensus was for ‘AA patients with a hair loss of more than 50% on the scalp can be considered as severe AA.’ and ‘AA patients who have significantly impaired QoL may be considered as moderate-to-severe AA regardless of hair loss percentage on the scalp.’ (Table 4).

**Table 4:** Results regarding AA disease severity perception and assessment.

Statements	Consensus %
Disease severity	
AA patients with a hair loss of 25–49% on the scalp can be considered moderate AA	82.5
AA patients with a hair loss of more than 50% on the scalp can be considered as severe AA.	100
Eyebrow, eyelash, and/or beard involvement in patients diagnosed with AA may be considered as moderate-to-severe AA regardless of hair loss percentage on the scalp.	95.6
Nail involvement in patients diagnosed with AA may be considered as moderate-to-severe AA regardless of hair loss percentage on the scalp.	81.7
AA patients who have significantly impaired QoL may be considered as moderate-to-severe AA regardless of hair loss percentage on the scalp.	100
Using systems that allow patients’ self-assessment of disease severity provides an ideal treatment approach by combining the patient’s and physician’s perspectives in AA management	86.9

Common observations, perspectives, and practices*	*%*
*Clinical practice*	
*I need a more practical scoring system (ex: less time-consuming, more inclusive of other factors rather than just scalp hair loss percentage) to assess the severity of the disease.*	*90.9*
*Disease severity scale/scoring systems are not used as much as they should evaluate AA patients in daily practice in my country.*	*95.4*

*More than 70% of the total participants and of the participants in each country. **Variation between countries, the total response is still over 70%**. AA, alopecia areata.

### 3.5 Treatment, disease flare, treatment response, and unmet need

The treatment section had the lowest consensus percentage, indicating significant differences among participants, including those from the same country, in their preferred treatment approach and duration. However, there was full agreement on certain literature-based statements, such as the primary goals of stopping further hair loss and promoting hair regrowth in cases where curative treatment is not possible, as well as the use of systemic corticosteroids only for temporarily halting disease progression in patients with rapidly progressing, widespread, active disease, all of which received a 100% consensus.

Other consensuses with the highest percentages were; ‘In the event that curative treatment is impossible, improving the QoL is also a primary goal of the AA treatment.’, ‘Topical calcineurin inhibitors (TCIs) have lower efficacy than topical corticosteroids in alopecia areata.’ and ‘JAK inhibitor (with or without SCS) can be initiated as a first-line systemic treatment in children over 12 years old with moderate-to-severe AA in whom the disease could not be controlled with optimal topical/local therapies. (From a scientific perspective assuming JAK inhibitor treatment is available and reimbursed for this condition for your patients.)’ (91.3, 91.3, 90.9%) (Table 5).

**Table 5:** Results regarding treatment preferences and management.

Item	Consensus %
Treatment	
The primary goal of the treatment in a patient diagnosed with AA is to achieve a cure.	95.6
In the event that curative treatment is impossible, stopping further hair loss and stimulating the regrowth of hair are primary goals.	100
In the event that curative treatment is impossible, improving the QoL is also a primary goal of AA treatment.	91.3
PUVA does not provide an optimal efficacy-safety balance in AA treatment; therefore, it should not be used.	72.7
Topical corticosteroids should be the first-line treatment irrespective of disease severity and disease phase in children up to 12 years of age.	73.8
Topical calcineurin inhibitors (TCIs) have lower efficacy than topical corticosteroids in alopecia areata.	91.3
Systemic corticosteroids should only be used to temporarily halt disease progression in patients with rapidly progressing, widespread, active disease.	100
Systemic corticosteroids (SCS) alone or in combination with local corticosteroids should be the first-line treatment for children over 12 years of age with active severe AA. (Systemic corticosteroids only as a temporary measure to contain rapidly progressing active disease).	72.7
SCS alone or in combination with local corticosteroids should be the first-line treatment for adults with active severe AA. (Systemic corticosteroids only as a temporary measure to contain rapidly progressing active disease).	81.8
ILC injections alone or in combination with local corticosteroids should be the first-line treatment for adults with active mild AA and/or AA with mild isolated patches of hair loss.	81.8
ILC injections alone or in combination with local/systemic corticosteroids should be the first-line treatment for adults with active moderate AA. (Systemic corticosteroids only as a temporary measure to contain rapidly progressing active disease).	81.8
ILC injections are more effective than ultrapotent/potent topical steroids for inducing regrowth and durable remission.	82.6
Topical minoxidil and topical anthralin can be used in between topical corticosteroids and topical immunotherapy	78.2
Topical immunotherapy should be the first-line treatment for children over 12 years of age with AA in chronic phases who do not respond o topical corticosteroid treatments regardless of disease severity.	81.8
Topical immunotherapy should be the first-line treatment for adults with AA in chronic phases who do not respond to topical corticosteroid treatments regardless of disease severity.	72.7
Steroid-sparing agents such as cyclosporine, AZA, and methotrexate should only be used to mitigate the risk of adverse effects associated with prolonged use of systemic corticosteroids.	73.9
JAK inhibitor (with or without SCS) can be initiated as a first-line systemic treatment in children over 12 years old with moderate-to-severe AA in whom the disease could not be controlled with optimal topical/local therapies. (From a scientific perspective assuming JAK inhibitor treatment is available and reimbursed for this condition for your patients.)	90.9
JAK inhibitor (with or without SCS) can be initiated as a first-line systemic treatment in adults with moderate-to-severe AA in whom the disease could not be controlled with optimal topical therapies. (From a scientific perspective assuming JAK inhibitor treatment is available and reimbursed for this condition for your patients.)	73.9
Efficient systemic treatment started at an early stage may prevent the development of disease-specific comorbidities in AA.	73.9

Common observations, perspectives, and practices*	*%*
*Patient journey insight items from different country settings*	
*The specialty/ies most commonly provide/s long-term follow-up for child patients with mild AA Dermatology.*	*86.9*
*The specialty/ies, most commonly provide/s long-term follow-up for child patients with moderate–severe AA; Dermatology.*	*100*
*The specialty/ies most commonly provide/s long-term follow-up for adult patients with mild AA: Dermatology.*	*100*
*The specialty/ies, most commonly provide/s long-term follow-up for adult patients with moderate to severe AA: Dermatology.*	
*AA is an under-treated disease in my country.*	*100*
*Clinical practice insight items from different country settings.*	
The treatment guidelines I follow to treat my AD patients: EADV**(Türkiye equally follows AAD).	*70.3*
Systemic corticosteroids (SCS) or Cyclosporin (CyC) with or without SCS are the first-line systemic treatments that I generally use for adult patients with moderate–severe AA despite optimal local/topical therapies** (Türkiye and Poland above 80%, other countries have different approaches within the country).	*72.7*
*Clinical response, duration of remission, and side effects are the most important factors which affect my systemic treatment preference in AA.*	*100*

*More than 70% of the total participants and of the participants in each country chose the same answer.

**Variation between countries, the total response is still over 70%. AA, alopecia areata; EADV, European Academy of Dermatology and Venereology; AAD, American Academy of Dermatology; SCS, Systemic Corticosteroids; CyC, Cyclosporine; ILC, intralesional corticosteroids.

Panelists stated that defining an AA flare is a complex process, and there is a need for standardization in defining measures of long-term disease control; it should be assessed by disease severity scores (such as SALT, AA-IGA, etc.) and can be defined as an episode requiring escalation of treatment (95.6%). Panelists fully agreed that potential adverse effects associated with long-term use and the risk of relapse on dose reduction or treatment cessation limit the use of all conventional systemic therapies for AA. Panelists also agreed on multiple treatment failure definitions; the highest rated ones are as follows; Inadequate clinical improvement despite appropriate dose and duration of and adherence to a therapeutic agent and failure to achieve stable long-term disease control despite appropriate dose and duration of and adherence to a therapeutic agent (86.9%). Current topical therapies, intralesional corticosteroids, and systemic immune response modifiers were not found to be sufficient by panelists to cover the therapeutic needs of patients with moderate-to-severe AA (95.6, 86.9, 91.3%) (Table 6).

**Table 6:** Results regarding disease flare, treatment response, and unmet need perceptions and observations.

Statements	Consensus %
Treatment response	
Defining an AA flare is a complex process, and there is a need for standardization in defining measures of long-term disease control.	95.6
Flares in AA can be defined as an increase in scalp hair loss noted by the patient or caregiver	86.9
AA flare can be defined as an episode requiring escalation of treatment	95.6
AA flare can be defined as an episode seeking additional medical advice.	82.6
Flare in AA patients should be assessed by disease severity scores (such as SALT, AA-IGA, etc.).	95.6
Inadequate clinical improvement despite appropriate dose and duration of and adherence to a therapeutic agent can be defined as treatment failure in AA.	86.9
Failure to achieve stable long-term disease control despite appropriate dose and duration and adherence to a therapeutic agent can be defined as treatment failure in AA.	86.9
Unacceptable adverse events or poor tolerability experienced with the treatment despite the appropriate dose and duration of and adherence to a therapeutic agent can be defined as treatment failure in AA.	82.6
The presence of ongoing impairment (e.g., depression, anxiety, and poor QoL) while on treatment despite appropriate dose and duration of and adherence to a therapeutic agent can be defined as treatment failure in AA.	77.2
Response to treatment in AA patients should be assessed by disease severity scores (such as SALT, AA-IGA, etc.)	86.9
There are currently no validated biomarkers that would predict response to treatment in AA patients.	95.6
There are no generally accepted criteria for defining treatment failure in AA.	91.3
Unmet Need	
Potential adverse effects associated with long-term use and the risk of relapse on dose reduction or treatment cessation limit the use of all conventional systemic therapies for AA.	100
Topical therapies (including immunotherapy) do not sufficiently cover the therapeutic needs of patients with moderate-to-severe AA.	95.6
Intralesional corticosteroids do not sufficiently cover the therapeutic needs of patients with moderate-to-severe AA.	86.9
The current systemic immune response modifiers do not sufficiently cover the therapeutic needs of patients with moderate-to-severe AA	91.3
There is a significant burden of adverse events with off-label use of currently available immunosuppressants in AA.	73.9
There is a significant unmet need for novel topical agents that offer prolonged remission and a safe side-effect profile in long-term moderate-to-severe AA treatment.	86.9
There is a significant unmet need for novel systemic agents that offer prolonged remission and a safe side-effect profile in long-term moderate-to-severe AA treatment.	86.9

Common observations, perspectives, and practices*	*%*
*Clinical practice*	
*I use disease severity scoring systems to assess flares in AA.*	*70.3*
*Observations*	
*Capturing AA flares in clinical practice through daily recording of medication use is feasible and appears to be a good indicator of long-term control.*	*72.7*
*Families of pediatric AA patients have more fears over systemic steroids’ side effects than topical steroids.*	*86.3*
*More than half of the adult patients with AA have systemic steroid phobia.*	*72.7*

*More than 70% of the total participants and of the participants in each country chose the same answer. SCORAD, scoring atopic dermatitis; AA, alopecia areata.

## 4 Discussion

The results of this Delphi study revealed that moderate-to-severe AA has a significant impact on the QoL of patients and their families from physician perspectives. This finding is consistent with several patient-reported outcome studies conducted in various countries, all of which confirmed that AA patients have significantly lower QoL, significant psychosocial burden compared to those without AA and that the severity of AA was associated with poorer QoL outcomes (8–11). QoL in AA patients was also shown to be significantly worse compared to other patients with cutaneous conditions, such as androgenetic alopecia and psoriasis (12, 13). In a survey study carried out in the United States, not only did children with AA experience diminished health related QoL (HRQoL), but their parents also manifested significant impairment. The adverse influence of AA is similarly observed in the partners of affected adults. Interestingly, parents indicated a more pronounced decline in HRQoL than their AA-affected children. The emotional strain on parents was found to intensify due to the absence of clinical improvement after consulting with multiple healthcare professionals, undergoing unsuccessful treatments, and incurring financial costs (14).

Furthermore, the panelists agreed that additional AA lesions in visible areas besides the scalp cause more significant impairment in patients’ QoL. Cross-sectional research undertaken in the United States identified a correlation between the loss of eyebrows/eyelashes and ocular discomfort/functional disruptions. Meanwhile, the absence of scalp hair was linked to heightened sensitivity to temperature fluctuations and an elevated potential for sunburns. This underscores the influence on QoL, extending beyond mere self-perception and societal stigmatization (14).

The panelists also agreed that AA significantly impacts work and productivity impairment in adults with moderate-to-severe conditions. In a UK-based population study from primary healthcare environments, individuals with AA were found to receive work leave certificates at a higher rate (13.0% within a year of diagnosis) compared to matched controls (7.9%) (aHR 1.56, 95% CI 1.43–1.71; *p* < 0.001). Additionally, those diagnosed with AA demonstrated a higher likelihood of being recorded as unemployed in the subsequent year after their diagnosis (1.3% of cases of AA, 0.6% of matched controls; aHR 1.82, 95% CI 1.33–2.49) (15). In a cross-sectional study involving 216 USA-based AA patients (of which 132 were employed), it was observed that 45% of those employed had taken time off work due to their AA condition (16). Another assessment involving dermatologists and their adult AA patients highlighted the existence of work productivity loss (WPL) and activity disruption stemming from AA-related emotional symptoms (ES) (17). In a separate survey focusing on workplace bullying in AA patients, it was found that 21.7% (*n* = 146) had encountered workplace bullying. The most common manifestations of such bullying were having their views overlooked (53.8%, *n* = 362), being ostracized (47.7%, *n* = 321), and being the subject of rumors (44.0%, *n* = 296). Intriguingly, 75.0% (*n* = 120/160) of those who self-identified as victims of bullying confronted the behavior, yet in 30.8% of these instances, the bullying persisted (30.8%, *n* = 37) (18).

While the diagnosis of AA is primarily clinical, there are several nuances and challenges that clinicians encounter in making an exact diagnosis. One of the primary challenges in diagnosing AA lies in its resemblance to other forms of nonscarring alopecia, such as trichotillomania, traction alopecia, and telogen effluvium. Therefore, despite the characteristic clinical features of AA, there are instances where additional investigations may be warranted to confirm the diagnosis or exclude other conditions (19, 20). As per the diagnostic approach in AA, this study reported that participant physicians agree that skin biopsies should be considered to exclude other conditions when the cause of hair loss is unclear. Numerous research articles have indicated that scalp biopsies enhance diagnostic precision compared to mere examination, potentially influencing both prognosis and therapeutic decisions (19, 20). The panelists agreed that trichoscopy is an essential tool for the assessment of disease activity and severity, whereas trichogram was only found to be a useful complementary tool for clinical evaluation, diagnosis, and monitoring of treatment as well as therapeutic monitoring. These results align with prior research which highlighted the efficacy of trichoscopy in diagnosing and tracking AA. Multiple other investigations have established trichoscopy as not only a beneficial instrument for assessing hair loss but also as surpassing the trichogram in its effectiveness (21, 22).

Moreover, literature suggest that the presence of associated autoimmune conditions, such as thyroid disorders and vitiligo, should prompt further evaluation to assess for underlying autoimmune polyglandular syndromes or immune dysregulation in suspected AA patients (23). Vast majority of study participants stated that they investigate autoimmune comorbidities (such as vitiligo, systemic lupus erythematosus, psoriasis, inflammatory bowel disease, thyroid disorders, ocular diseases and rheumatoid arthritis.) in their adult and pediatric patients with moderate to severe AA (86.9%).

Negative prognostic factors associated with alopecia areata (AA) extend beyond autoimmune comorbidities, with emerging evidence suggesting correlations with atopy. Atopic dermatitis (AD), a chronic inflammatory skin condition characterized by pruritus and eczematous lesions, shares immunological pathways with AA, potentially influencing disease outcomes. Studies have reported increased prevalence of AD among AA patients, indicating a possible association between the two conditions (24). Furthermore, shared genetic susceptibility loci and dysregulated immune responses, particularly involving T-helper cell subsets and cytokine profiles, may contribute to the co-occurrence of AA and AD. Clinical observations suggest that patients with concurrent AA and AD may experience more severe disease manifestations and treatment challenges, highlighting the need for comprehensive management strategies.

AA patients with a hair loss of more than 50% on the scalp were agreed to be considered as having a severe AA. However, the practicality of existing severity indices, such as the Severity of Alopecia Tool (SALT) and Alopecia Areata Investigator Global Assessment (AA-IGA), remains questionable due to their limited sensitivity and applicability in routine clinical practice. The panelists also underlined the need for a more practical scoring system to assess the severity of AA. Existing scoring systems were not found sensitive enough to reflect AA’s complex and heterogeneous nature in other studies. Furthermore, the panelists reported that disease severity scales are not widely used in daily practice to evaluate AA patients in their countries. Indeed, studies from both the US and Europe have highlighted the inconsistent use of severity scales in daily patient evaluation, reflecting the need for more standardized and objective assessment tools (25, 26). Efforts to bridge the gap between research methodologies and clinical reality are imperative to enhance patient care. A holistic approach that integrates patient-reported outcomes, clinician assessments, and objective measures may offer a more comprehensive understanding of AA severity and treatment response in real-world settings.

The Delphi consensus study provided valuable insights into the management of AA, particularly in defining an AA flare, long-term disease control, and treatment failure definitions. Panelists agreed that defining an AA flare is complex, and there is a need for standardization in defining measures of long-term disease control. Severity indices, like SALT and AA-IGA, have been identified as effective instruments for gauging disease intensity and managing flare-ups. Research from both the US and Europe further emphasized the significance of these severity metrics in appraising AA patients and overseeing treatment outcomes, corroborating the consensus of the panelists (27, 28).

Regarding the treatment of moderate-to-severe AA, the panelists agreed that current therapies, including topical treatments, intralesional corticosteroids, and systemic immune response modifiers, are insufficient to cover patients’ therapeutic needs. Although numerous studies have demonstrated that topical minoxidil can promote new hair growth, using it as a standalone therapy yielded limited results. Specifically, hair growth was only observed in cases of mild AA, while it was ineffective for severe AA (29). On the other hand, topical immunotherapy employing diphenylcyclopropenone (DPCP) was proven to be successful in several research, promoting hair regrowth. However, there’s a considerable risk of side effects and relapse upon long-term observation (30, 31). A systematic review pinpointed systemic corticosteroids as effective in promoting hair regrowth among patients with severe AA, yet they come with substantial risks of side effects, including diabetes and osteoporosis (29). Furthermore, the scientific literature lacks a unified stance on the recommended dosage and duration for the daily use of systemic corticosteroids in AA treatments.

Moreover, the panelists acknowledged the significant burden of adverse events associated with the off-label use of currently available immunosuppressants, highlighting the importance of developing new therapies that offer a safe side-effect profile on long-term treatment. Expert opinion studies based on available evidence suggests that Cyclosporine is not a favored choice for AA due to its pronounced side-effect profile and considerable relapse rate. Such side effects encompass nephrotoxicity, immunosuppression, hypertension, and excessive body hair growth, known as hypertrichosis (32). Furthermore, the use of oral methotrexate for alopecia areata has been linked to severe adverse events, including gastrointestinal issues and liver toxicity (33). All available evidence strongly emphasizes the need for developing new therapies that promise enhanced safety and more acceptable tolerability for AA patients.

Recent studies have reported promising results in using Janus kinase inhibitors (JAK inhibitors) for the treatment of AA (34–39). A recent systematic review and meta-analysis, encompassing 7 randomized clinical trials and involving 1710 patients, demonstrated that JAK inhibitors were more efficacious in promoting hair regrowth than placebos. Notably, the effectiveness of oral JAK inhibitors appeared superior to topical applications, and there was no observed increase in treatment-related adverse events (AEs) when compared to placebos (40). Baricitinib is approved for use only in adult severe AA patients, whereas ritlecitinib is approved for the same use in people ages 12 years and older. While JAK inhibitors offer promising therapeutic benefits and now in clinical use, concerns regarding their safety profile remain. The most common adverse effects due to oral JAK inhibitors were upper respiratory tract infections, urinary infections, headache, laboratory abnormality, and acne. These side-effects are similar to those reported in previous reviews of JAK inhibitors in patients with alopecia. Laboratory abnormalities included cytopaenias, lipid abnormalities, and an increase in blood creatine phosphokinase. No cases of reactivation of tuberculosis or new malignancies were reported (41). Despite serious adverse effects being less reported in AA studies, it is important to highlight that data regarding the safety of JAK inhibitors in relation to AA is still in its infancy. Data from clinical trials have underscored the importance of careful patient selection, particularly in individuals with cardiovascular comorbidities, to mitigate potential risks associated with JAK inhibitor therapy. Moreover, proactive measures such as herpes zoster vaccination may serve to minimize the risk of opportunistic infections in patients undergoing treatment with JAK inhibitors.

The high cost of JAK inhibitors may limit their accessibility to some patients. Therapeutic challenges in alopecia areata (AA) may present unique considerations especially in Central Eastern Europe, Türkiye or Russia, reflecting regional variations in healthcare access, treatment availability, and patient preferences. While conventional treatments such as topical corticosteroids and systemic immunosuppressants are commonly used, challenges may arise due to limited access to newer therapies like Janus kinase inhibitors (JAK inhibitors). Despite both FDA approved JAK inhibitors are also approved by EMA, they are still not reimbursed in some CEE countries or available through self-funding for AA. Studies suggest that economic factors and regulatory barriers may impact the adoption of innovative treatments, potentially affecting treatment outcomes. Additionally, cultural perceptions of hair loss and dermatological conditions may influence patient attitudes toward seeking medical care and adherence to treatment regimens (42). Collaborative efforts between healthcare providers, policymakers, and patient advocacy groups are essential to address these challenges and improve the management of AA in the region.

In conclusion, the findings of this Delphi study demonstrate a significant disease burden and unmet medical need for patients with moderate-to-severe AA from the perspective of experts. These findings are consistent with previous studies conducted in different countries, highlighting the need for and importance of providing patients access to recently approved novel treatments and for further research to develop more effective therapies with tolerable safety profile for AA.

## 5 Limitations

The limitations of this study are inherent in the nature of the Delphi method. One limitation is the small number of experts and differences in the representation of each country, which may not fully reflect the approaches and insights of the majority of experts at an ideal level. Additionally, the absence of AA patients on the panel limits the ability to reflect their perspectives on the disease burden and unmet treatment needs. The delay between 2 rounds might have an effect on the validity of participants’ current opinion, however none of the participants objected nor wanted to revise any claims on these outcomes. Additionally, this Delphi study also enabled a comprehensive and systematic exploration of clinical, diagnostic, and follow-up approaches in patients with AA based on the expert opinions of qualified dermatologists in Poland, Czechia, Russia, and Türkiye within a limited time frame. The findings and limitations of this study can serve as reference points to initiate and establish more focused and populated consensus studies not only in these countries but also in other developing countries.

## Data availability statement

The original contributions presented in the study are included in the article as well as in supplementary material which can be obtained from the corresponding author.

## Author contributions

LR: Conceptualization, Data curation, Formal analysis, Investigation, Methodology, Project administration, Supervision, Validation, Writing – review & editing. MT: Investigation, Validation, Writing – review & editing. EA: Validation, Writing – review & editing. PA: Validation, Writing – review & editing. SA: Validation, Writing – review & editing. NB: Validation, Writing – review & editing. SB: Validation, Writing – review & editing. MB: Validation, Writing – review & editing. MC: Validation, Writing – review & editing. BE: Validation, Writing – review & editing. TE: Validation, Writing – review & editing. IS: Validation, Writing – review & editing. OF: Validation, Writing – review & editing. AG: Validation, Writing – review & editing. MK: Validation, Writing – review & editing. AL: Validation, Writing – review & editing. AM: Validation, Writing – review & editing. NM: Validation, Writing – review & editing. NO: Validation, Writing – review & editing. WO: Validation, Writing – review & editing. ZP: Validation, Writing – review & editing. AR: Validation, Writing – review & editing. MS: Validation, Writing – review & editing. BG: Validation, Writing – review & editing.
